# Monitoring performance of sites within multicentre randomised trials: a systematic review of performance metrics

**DOI:** 10.1186/s13063-018-2941-8

**Published:** 2018-10-16

**Authors:** Kate F. Walker, Julie Turzanski, Diane Whitham, Alan Montgomery, Lelia Duley

**Affiliations:** Nottingham Clinical Trials Unit, QMC, Nottingham, NG7 2UH UK

**Keywords:** Multicentre, Randomised trials, Clinical trials, Performance metrics, Trial management, Site performance, Operational metrics, Key performance indicators

## Abstract

**Background:**

Large multicentre trials are complex and expensive projects. A key factor for their successful planning and delivery is how well sites meet their targets in recruiting and retaining participants, and in collecting high-quality, complete data in a timely manner. Collecting and monitoring easily accessible data relevant to performance of sites has the potential to improve trial management efficiency. The aim of this systematic review was to identify metrics that have either been proposed or used for monitoring site performance in multicentre trials.

**Methods:**

We searched the Cochrane Library, five biomedical bibliographic databases (CINAHL, EMBASE, Medline, PsychINFO and SCOPUS) and Google Scholar for studies describing ways of monitoring or measuring individual site performance in multicentre randomised trials. Records identified were screened for eligibility. For included studies, data on study content were extracted independently by two reviewers, and disagreements resolved by discussion.

**Results:**

After removing duplicate citations, we identified 3188 records. Of these, 21 were eligible for inclusion and yielded 117 performance metrics. The median number of metrics reported per paper was 8, range 1–16. Metrics broadly fell into six categories: site potential; recruitment; retention; data collection; trial conduct and trial safety.

**Conclusions:**

This review identifies a list of metrics to monitor site performance within multicentre randomised trials. Those that would be easy to collect, and for which monitoring might trigger actions to mitigate problems at site level, merit further evaluation.

## Background

Multicentre randomised trials are complex and expensive projects. Improving the efficiency and quality of trial conduct is important, for patients, funders, researchers, clinicians and policy-makers [1]. A key factor in successful planning and delivery of multicentre trials is how well sites meet their targets in recruiting and retaining participants, and in collecting high-quality, complete data in a timely manner [2]. Collecting and monitoring easily accessible data relevant to performance of sites has the potential to improve the efficiency and success of trial management. Ideally, such performance metrics should provide information that quickly identifies potential problems so they can be mitigated or avoided, hence minimising their impact and improving the efficiency of trial conduct.

We are not aware of any standardised metrics for monitoring site performance in multicentre trials. A recent query to all UK Clinical Research Collaboration (UKCRC), registered Clinical Trials Units (CTUs) revealed that many units routinely collect and report data for each site in a trial; such as numbers randomised, case report forms (CRFs) returned, data quality, missing primary outcome data, and serious breaches. How such data are used to assess and manage performance varies widely however [3–7]. Agreeing a small number of metrics for site performance that could be easily collected, presented and monitored in a standardised way by a trial manager or trial co-ordinator would be a potentially useful tool to improve efficient trial conduct.

Currently, trial teams, sponsors, funders and oversight committees monitor site performance and trial conduct based primarily on recruitment [8]. Whilst clearly important, recruitment is not the only performance indicator that matters for a successful trial. Using a range of additional metrics that include data quality, protocol compliance and participant retention would give a better overall measure of the performance of each trial site, and the trial overall. To be low cost and efficient, the number of metrics monitored at any one time should be limited to no more than 8 to 12 [9]. We conducted a systematic review to identify performance metrics that have been used, or proposed, for monitoring or measuring performance at sites in multicentre randomised trials.

## Methods

We performed a systematic review to identify metrics that have been used or proposed for monitoring or measuring performance at individual sites in multicentre randomised trials.

### Criteria for potentially eligible studies

Studies were potentially eligible for inclusion if they:

- Reported one or more site performance metric, either used or proposed for use, specifically for the purpose of measuring individual site performance
- Were multicentre randomised trials, or concerning multicentre trials
- Were published in English
- Related to randomised trials involving humans

Studies where the strategy for monitoring site performance was randomly allocated were included. We anticipated that there might be studies where the adoption of an individual performance metric might have been tested by randomly allocating sites to using that particular metric or not. Studies relevant to both publically funded and industry-funded trials were included.

### Search strategy

We searched the Cochrane Library and five biomedical bibliographic databases (CINAHL, Excerpta Medica database (EMBASE), Medical Literature Analysis and Retrieval System Online (Medline), Psychological Information Database (PsychINFO) and SCOPUS) and Google Scholar from 1980 to 2017 week 07. The search strategy is provided as an Appendix (Table 3).

### Selection of studies

Two reviewers (KW, JT) independently assessed for inclusion the titles and abstracts identified by the search strategy. If there was disagreement about whether a record should be included, we obtained the full text.

We sought full-text copies for all potentially eligible records, and two reviewers (KW, JT) independently assessed these for inclusion. Disagreements were resolved by discussion, and if agreement could not be reached the study was independently assessed by a third reviewer (LD). Multiple reports of the same study were linked together.

### Data extraction and data entry

Two reviewers (KW, JT) extracted data independently onto a specifically designed data extraction form. In the few cases where full text was not available (*n* = 9), data were extracted using the title and abstract only. Data were entered into an Excel spreadsheet, and checked.

Data were extracted on the design of the randomised trial (participants, intervention, control, number of sites and target sample size); whether the performance metric/s was theoretical or applied. For each performance metric we collected data that included: a verbatim description of the metric; how the metric was measured or expressed; timing of the measurement and during which phase of the study; who measured the metric; if a threshold exists to trigger action, what the threshold was and what action it triggers; and whether the metric was recommended by the authors.

### Data analysis

We described the flow of studies through the review, with reasons for being removed or excluded, using the Preferred Reporting Items for Systematic Reviews and Meta-Analyses (PRISMA) guidance [10]. Characteristics of each study were described and tabulated. Analyses were descriptive only, with no statistical analyses anticipated.

## Results

**Fig. 1:**
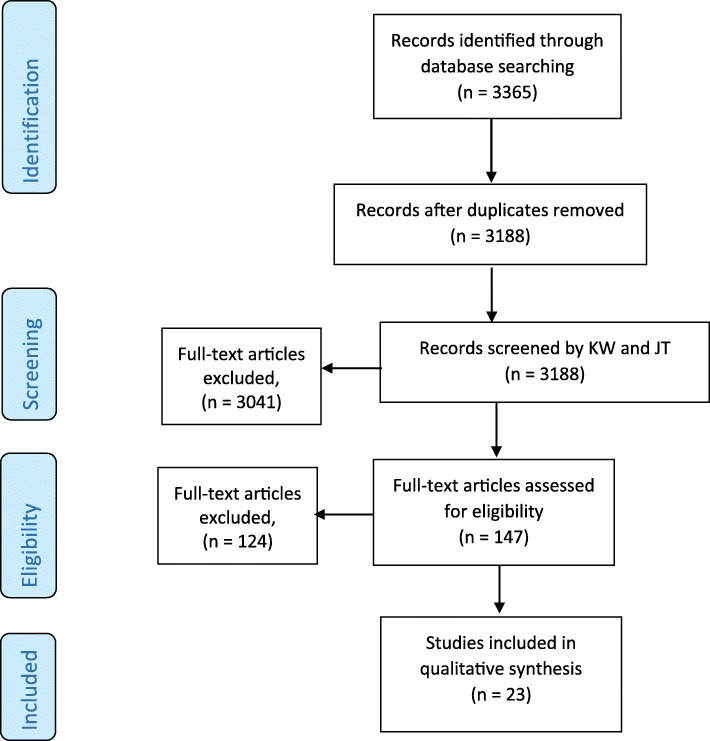
Flow diagram

The database search identified 3365 records, of which 177 were duplicates, leaving 3188 screened for eligibility (Fig. 1). At screening, we obtained full-text copies for 147 records to determine eligibility. For a further seven records full-text copies were unavailable, and so screened was based on the abstract only. Of those full-text copies and abstracts (for papers where the full text was unavailable), there was disagreement on three papers. Following discussion two papers were accepted for inclusion [11, 12] and one paper was excluded [13].

Twenty-one studies were agreed for inclusion, of which 14 were studies proposing performance metrics and seven were studies using performance metrics (Table 1). These 21 studies reported a total of 117 performance metrics. The median number of performance metrics reported per study was 8, with the range being 1–16. Those 117 metrics were then screened, to exclude any judged as: lacking sufficient clarity; being unrelated to individual site performance; being too specific to an individual trial methodology or pertaining to clinical outcomes not trial performance. This left 87 performance metrics to be considered for use in day-to-day trial management. The metrics broadly fell into six main categories: assessing site potential before recruitment starts; and monitoring recruitment, retention, quality of data collection, quality of trial conduct, and trial safety (Table 2).

**Table 1:** Characteristics of included studies

Study	Study description	Number of sites (sample size)	Metrics reported by each study	
			Included as site performance metric	Excluded as not site performance metric^a^
Studies proposing performance metrics				
Bose 2012 [14]	Paper discussing trial management through central monitoring	Not applicable	• Site location potential index based on an assessment of the number of patients at an individual site with the disease of interest • Trial compliance index based on a number of suggested factors including the number of late visits, failure to achieve recruitment target, number of dosing errors, etc.	• Drug adversity measurement (B) • Drug potential index (B)
Djali 2010 [15]	Paper discussing a data-driven quality management system	Not applicable	• Enrolment number per site^b^ • Recruitment period per site • Number of AEs per site • Number of protocol deviations and violations per site	• Number of discontinuations per site (A) • Deaths per site (D)
Elsa 2011 [16]	Methodology of developing ‘key risk indicators’ for monitoring of a large international clinical trial	Not applicable	• Rate of SAE reporting per site: centres assigned a dichotomous score depending on whether they showed extreme deviation from comparable sites (arbitrarily defined as half the observed median rate across sites) • Short visit duration: centres assigned a dichotomous score depending on whether they showed extreme deviation from comparable sites (arbitrarily defined as half the observed median rate across sites)	• Measures of compliance with study treatment (A) • Blood results/other continuous variables examined for unusual patterns (A)
Glass 2007 [17]	Study analysing data retrospectively from 262 clinical trials to determine variables associated with successful trial delivery	Not applicable	• Actual number participants randomised per site • Number successfully completing the study’s protocol per site • Time between when an individual site randomises its first participant and the time the first site in that study enrols its first patient	
Hanna, 2013 [11]	Development of a list of quality indicators for trial performance based on the consensus of experts	Not applicable	• SAE reporting measured by the number of SAEs reported/number of SAEs identified in trial database or trial follow-up documents • Transfer of CRF to CTU measured by the number of completed CRF received by CTU within 30 days/number of completed CRF received by the CTU in 3 months	
Jou, 2013 [18]	Aim of the main study: treatment-naïve, hepatitis C patients randomised to two peginterferon regimens. Primary outcome virologic response. A retrospective analysis was performed of individual site performance using trial data	118 (3070)	• Rates of screen failure defined as the percentage of participants screened who failed screening • Completion and discontinuation of treatment, defined as the percentage of participants who completed treatment/ percentage of participants who discontinued treatment • Completion / discontinuation of follow-up, defined as the percentage who completed follow-up/ percentage who discontinued follow-up	• Treatment adherence (B)
Khatawkar 2014 [19]	Retrospective analysis of data queries using clinical trial data	Not applicable	• Data query (DQ) rate per page • DQ rate per page by phase of study	• DQ rate per page by country (B) • DQ rate per page by therapeutic area (B)
Lee 2012 [20]	Paper describing the output of a Delphi survey to establish an ‘evaluation framework’ for clinical trial data	Not applicable	• Rapid enrolment, defined as time taken to reach target enrolment • Timely data entry, defined as time taken for data entry after completion of informed consent • Timely manual query management, defined as time taken for response to manual query request from data centre • Timely database lock, defined as time taken for database lock after the last visit of last participant per site • Data discrepancy management metric encompassing number of manual queries per CRF for missing data; number of manual queries per CRF for out -of-range data; number of manual queries per CRF for logical consistency • Protocol compliance metric encompassing: rate of ‘dropout’ of total participants; rate of false ‘dropout’ of total dropouts; rate of late detection of ‘dropout’ • Enrolment success defined as % eligible per study	• Weeks after go-live, i.e. after the point of protocol amendment (A)
Rojavin, 2005 [21]	Paper describing and discussing one proposed metric	Not applicable	• Recruitment Index (RI) = (LPFV − FPFV) x S/P where LPFV = date of the last participant first visit FPFV = date of the first participant first visit S = number of participating sites P = number of participants who successfully completed the study	
Rosendorf, 1993 [22]	Trials of treatment for HIV. No further details. An evaluation tool was proposed to monitor individual site performance within a multicentre randomised trial.	59 (ns)	Intensity adjusted score (IAS) = IAS = IS0 + don x IS1 + doff x IS2 where: IS0 = score assigned for enrolling a new participant during the 6 month evaluation period don = number of days the participant was on the study medication during the evaluation period doff = number of days the participant was off the study medication IS1 = intensity score for the days in which the participant is receiving study medication IS2 = intensity score for the days in which the participant is off all study medication ISA is calculated for each participant and then summing scores across all participants, once during the evaluation period • Funding adjusted score = IAS divided by the amount awarded for total direct costs during the given time period • Summary quartiles = total number of new and continuing participants on study	
Sweetman, 2011 [23]	Retrospective analysis of publications of 80 clinical trials on protocol violations reporting	Not applicable	Occurrence of protocol violations, defined as total number of protocol violations divided by the number of enrolled participants	
Thom, 2011 [12]^a^	Report of a centre performance assessment tool used within a clinical trial network to assess individual site performance	Not applicable	• Protocol adherence, defined as average rate of protocol violations per enrolled participant • Data quality, defined as average rate of edit checks per participant • Data timeliness, defined as the percentage of forms entered late • Time of starting after the first centre start date • Sum of protocol adherence, data quality, data timeliness and timeliness of study start-up to give overall rank • Timeliness of study start-up	• Recruitment, defined as average percentage of participants contributed over all studies conducted (B) • Retention, defined as average percentage of participants with complete follow-up data (B) • Recruitment/retention, defined as sum of recruitment + retention to give overall rank (B) • Adherence/quality (A) • Quality of laboratory samples collected (A)
Tudur Smith, 2014 [24]	Paper describing monitoring methods using a ‘risk proportionate approach’ used by an individual clinical trials unit	Not applicable	• Consent form completion, defined as consent forms returned within 7 days of completion by sites. • Recruitment process, defined as frequency of eligible participants who do not provide consent. • Missing primary outcome data, defined as cumulative percentage of participants with missing primary outcome data at each site • SAEs, defined as cumulative percentage of participants with at least one SAE across the trial as a whole and at each site /measure of time, e.g. 1 month • Sum of all SAEs/sum of all follow-up for the trial • Sum of all follow-up at site x overall SAE rate for the trial • Visit dates, defined as time between actual date of visit versus expected date of visit	• Case report form completion, defined as timely submission (A)
Wilson, 2014 [25]	Theoretical paper describing methods of monitoring the conduct of trials	Not applicable	• Quality metric encompassing: average number of major audit findings per audited site; percentage per site of unreported, confirmed SAEs; number of significant protocol deviations per site • Frequency of protocol violations for eligibility criteria and randomisation per site • Rates of withdrawal by site	• Proportion of the enrolled population comprising the non-randomised parallel cohorts (measured by percentage agreement and kappa statistic) (C) • Radiologic inter-observer agreement (C)
Studies using performance metrics				
Berthon-Jones 2015 [2]	Aim of main study: treatment-naïve HIV patients randomised to 2 different types of ART. Primary outcome plasma HIV-RNA, change from baseline to week 48. Performance across 5 geographical regions was assessed using performance metrics	36 (322)	• Time from protocol release to ethics/regulatory submission • Time from protocol release to ethics/regulatory approval • Time from protocol release to first participant randomised (FPR) • Time from protocol release to last participant randomised (LPR) • Time from site opened to first participant randomised (FPR) • Time from first participant randomised (FPR) to last participant randomised (LPR) • Actual versus estimated recruitment • Time from participant visit to electronic data capture (EDC) initiation • Time from EDC initiation to completion • Number of missing values per participant • Number of data queries per participant • Number of SAEs reported per participant • Time from SAE occurrence to initial report • Time from initial SAE report to final report • Number of samples collected versus number required by protocol	• Number of missed visits per region (B) • Quality of laboratory sample/s collected (A) • Number of plasma samples collected versus protocol-mandated samples to be collected (C) • Number of buffy-coat samples collected versus protocol-mandated samples to be collected (C)
Katz, 2015 [26]	Aim of main studies: osteoarthritis (2 trials), lower back pain (1 trial) randomised to fulranumab infusion or placebo. Primary outcomes unspecified. Within these three clinical trials a method of monitoring individual site performance was applied	40–88 (91–157)	• Time to data query response	• Compliance with study drug (D)
Kim, 2011 [27]^a^	Aim of main study: patients with acute cerebral haemorrhage randomised to early intensive antihypertensive or standard regimen. Primary outcome death or disability at 3 months. A site performance monitoring tool was incorporated for monitoring individual site performance during the trial	100 (1280)	• Participant recruitment per site • CRF data collection timeliness + completeness • Protocol violations per site • SAE reporting per site	• Participant study progress (A) • Site data monitoring visit findings (A) • Data clarification request processing (A) • Regulatory document collection and tracking (A)
Rifkind, 1983 [28]	Aim of the main study: men with primary type 2 hyper-lipoproteinaemia randomised to bile acid sequestrant or placebo. Primary outcome CHD death and/or nonfatal myocardial infarction. Within this study measures of individual site recruitment performance were monitored.	12 (3550)	• Proportion of initial contacts proceeding to first protocol visit by recruitment source • Proportion of first protocol visits proceeding to study entry by recruitment source	
Saunders, 2015 [29]^a^	Aim of the main study: critical care patients randomised to probiotic or placebo. Primary outcome ventilator associated pneumonia. Within this study the team focused on screening performance in individual centres	14 (285)	• Non-screening weeks = proportion of weeks during which participants were not screened for trial eligibility	•
Sun, 2008 [30]	Aim of the main study: patients with major depression randomised to aprepitant or placebo. Primary outcome change in Hamilton Depression Scale. Within this study measures of individual site performance were captured	Not reported	• Administration excellence, defined as site administration performance and interaction with central study team rated 1, 2 or 3 • Data quality, defined as data completeness and correctness at initial submission rated 1, 2, or 3 • Proportion of participants with protocol violation, defined as: proportion of participants in each site who do not meet eligibility criteria; have medication compliance < 75%, or take prohibited concomitant medication or wrong study medication; or other serious violation • Level of visit non-compliance, defined as mean absolute difference of the days between visits and the protocol-specified days between visits for participants in a specific centre	• Level of medication non-compliance, defined as the mean percentage of days participants from each centre taking less than the prescribed number of doses of study-assigned medication (B)
Wear, 2010 [31]^a^	Aim of the main study: patients with multiple myeloma, multiple clinical trials. No further details. Performance metrics utilised during the study	Not reported	• First patient dosed (FPD), defined as time from receipt of final protocol to the first participant treated • Enrolment commitment (EC), defined as commitment from the study site to provide a predicted number of participants who will receive at least 1 dose of study drug (e.g. number of participants randomised and completing first part of intervention • Baseline enrolment timeline (BET), defined as target time period to obtain EC	

*AE* adverse event; *ART* antiretroviral therapy; *CHD* coronary heart disease; *CRF* case record form; *CTU* clinical trial unit; *ns* not specified; *SAE* serious adverse event; *VTE* venous thromboembolism

^a^Excluded due to (a) lack of clarity, (b) not related to individual site performance, (c) too specific to an individual trial methodology, (d) pertaining to clinical outcomes not trial performance

^b^It is unclear from the paper whether enrolment refers to participants randomised to a study or simply consented and then screened for study eligibility

**Table 2:** Examples of performance metrics within each identified category

Categories	Example performance metric	Studies in which metric included
Assessing site potential	Site location potential index based on an assessment of the number of patients at an individual site with the disease of interest	[14]
Monitoring recruitment	Number of participants randomised per site	[15, 17, 27]
Monitoring retention	Rates of withdrawal by site	[20, 25]
Quality of data collection	Number of data queries per participant	[2, 12, 19]
Trial conduct	Protocol violations per site or per participant	[12, 15, 23, 27, 30]
Trial safety	Serious adverse event (SAE) reporting per site	[11, 24, 27]

## Discussion

As far as we are aware, this is the first systematic review to identify and describe proposed or utilised metrics to monitor site performance in multicentre randomised trials. It provides a list of performance metrics, which can be used to contribute to developing and agreed a proposed set of performance metrics for use in day-to-day trial management. We identified 87 performance metrics which fell broadly into six main categories.

A strength of our study was the comprehensive search of the literature.

In planning this systematic review we envisaged that studies would be identified that had evaluated individual performance metrics either by implementation mid-way through a study, or ideally by randomising individual sites to use of a particular metric or not. Unfortunately, there was a paucity of such studies. Most studies suggested performance metrics on a purely theoretical basis, and did not provide data on the actual use of suggested metrics. The main limitations of our study were the lack of studies implementing performance metrics and reporting the effects of their utilisation, and that published work on this topic is limited, which is perhaps surprising as informal assessment of how sites perform in multicentre trials is common.

This list of performance metrics contributed to development of a Delphi survey sent to trial managers, UKCRC CTU directors and key clinical trial stakeholders, which is reported elsewhere. They were invited to participate through the UK Trial Managers’ Network (UK TMN) and UK Clinical Research Collaboration (UKCRC CTU) Network. Three Delphi rounds were used to steer the groups to consensus, refining the list of performance metrics. The reasons for their decisions were documented. Finally, data from the Delphi survey was presented to stakeholders in a priority setting expert workshop, providing participants with the opportunity to express their views, hear different perspectives and think more widely about monitoring of site performance. This was used to establish a consensus among experts on the top key performance metrics, expected to number around 8–12.

## Conclusions

This study provides trialists for the first time with a comprehensive description of performance metrics described in the literature that have been proposed or used in the context of multicentre randomised trials. It will assist future work to develop a concise, practical list of performance metrics which could be used in day-to-day trial management to improve the performance of individual sites. This has the potential to reduce both the financial cost of delivering a multicentre trial, and the research waste and delay in scientific progress that results when trials fail to meet their recruitment target, are poorly conducted, or have inadequate data.

## Appendix

**Table 3:** Search strategy. Monitoring performance of sites within multicentre randomised trials: a systematic review of performance metrics

#	Searches
1	Randomised. controlled trial
2	Clinical trial
3	Pragmatic trial
4	Controlled clinical trial
5	1 or 2 or 3 or 4
6	Performance indicator
7	Performance metric
8	Performance measure
9	Enrollment rate
10	Participant enrollment
11	Participant recruitment
12	Quality indicator
13	Quality measure
14	Performance management
15	Assessing site performance
16	Central monitoring
17	Clinical trial monitoring
18	Clinical trial reporting
19	Trial analytics
20	Trial management
21	Site performance
22	Study conduct
23	Trial site performance
24	Benchmarking performance
25	Clinical data management
26	Clinical trial data quality
27	Laboratory sample quality in clinical trials
28	Operational metrics
29	Operational performance
30	Performance evaluation
31	Performance monitoring
32	Performance score
33	Protocol deviations
34	Protocol violations
35	Quality management system
36	Recruitment index
37	Screening logs
38	Strategic project management
39	6 or 7 or 8 or 9 or 10 or 11 or 12 or 13 or 14 or 15 or 16 or 17 or 18 or 19 or 20 or 21 or 22 or 23 or 24 or 25 or 26 or 27 or 28 or 29 or 30 or 31 or 32 or 33 or 34 or 35 or 36 or 37 or 38
40	39 and 5
41	40 Not (animals/ not humans.sh.)
42	40
43	Limit 42 to English language

## Abbreviations

CINAHL: Cumulative Index to Nursing and Allied Healthy Literature
CRF: Case report form
CTUs: Clinical Trials Units
EMBASE: Excerpta Medica database
Medline: Medical Literature Analysis and Retrieval System Online
NIHR: National Institute for Health Research
PRISMA: Preferred Reporting Items for Systematic Reviews and Meta-Analyses
PsychINFO: Psychological Information Database
UK TMN: UK Trial Managers’ Network
UKCRC: UK Clinical Research Collaboration
